# Regulatory bodies must critically assess their role in the wellness wave of supplement marketing

**DOI:** 10.1016/j.lana.2025.101348

**Published:** 2025-12-26

**Authors:** Alessandro R. Marcon

**Affiliations:** Health Law Institute, Faculty of Law, University of Alberta, Office 470, Edmonton, T6G 2H5, Alberta, Canada

Over the past decades, wellness has ballooned into a multitrillion-dollar industry.^1^ Defined multifariously across physical, mental, spiritual, emotional, occupational, environmental, and social dimensions, wellness is portrayed and realised through activities to maintain and optimise oneself in relation to ever-shifting ideals.^2^ Supplements or natural health products (hereafter “*supplements*”) form a key component and commercial staple of these wellness ideals.^1^^,^^2^ In turn, high supplement use is generating complex regulatory challenges. Challenges concern product safety but increasingly relate to pernicious social issues around public perspectives of the health sciences and government roles in public health.

Supplements are diversely defined and categorised across jurisdictions, broadly encompassing vitamins, minerals, herbs or botanicals, amino acids, and pre/probiotics.^3^ Already presenting high-frequency public use and ubiquitous marketplace presence, supplement use skyrocketed during the COVID-19 pandemic, with consumers seeking purported immunity-strengthening benefits.^4^ Research has shown that immune-boosting health claims circulated uncritically online during the pandemic,^5^ and has detailed an emerging online marketplace of “stem cell” supplements, whose marketing promotes immunity-improving and anti-ageing benefits.^6^ Supplement use is prevalent among cancer patients,^7^ and athletes seeking performance enhancements.^8^ In controversial contexts, supplement regimens are being promoted and sold as ALS treatments.^9^ There is, therefore, growing concern that supplements are not merely sought out as supplements to dietary deficiencies but as key preventative tools, enhancement aids, and therapeutics to address potentially serious ailments. It is on this basis that regulatory challenges are intensifying.

Aside from established issues around supplement quality and labelling accuracy,^4^ the crux of the public health issue centres on health claims. Worldwide, supplement regulation works on the basis that supplements differ from medicines, being low risk and containing no novel substances.^3^ Supplement health claim regulation in the EU, New Zealand, and Australia works towards ensuring that product claims align with this profile.^3^ Similarly, in Canada and the US, regulation prohibits supplement marketing with direct causal claims (e.g. “cure”, “treat”, “prevent”) for “serious” ailments.^6^ Yet this same marketing allows, and arguably enables—in the form of structure/function claims (U.S.) and general health claims (Canada)—marketing which describes indirect, vague and supportive mechanisms of action for bodily functions and general ailments.^6^ In the case of “stem cell” supplement marketing on Amazon, for example, products do not claim benefit for “dementia” but do for “ageing,” “memory,” “cognitive functioning,” and “brain fog.”^6^ The dangerous presumption is that laypeople are able to accurately interpret this marketing as products with indirect, marginal, or inconclusive effect. While regulation states that marketing cannot distort or manipulate the relevant evidentiary consensus, research shows the common presence of this very discourse.^4^^,^^6^

The greatest harm from widespread supplement use is thus arguably not the negative impacts from individual products but instead how marketing distorts what supplements can do, and as a result, undermines the ongoing scientific evidentiary base for efficacious interventions, like vaccines. Further, wellness discourse promotes the idea that supplement use is integrally part of self-care, creating a moral impetus, or even burden, to properly and assiduously care for oneself.^2^ Critically, supplement marketing does not discursively exist in a vacuum of individual advertisements. It intertwines with overarching wellness ideas, which, while promoting alternative products, can connote ideas that modern medicine strives to keep people sick, that healthcare withholds cures from the public, that vaccines aren't necessary, don't work, or kill, and that scientific processes enabling therapeutic development are solely profiteering enterprises, unconcerned with effective public health outcomes. It thus often creates fear and mistrust around established science and scientists, including established regulatory bodies.

The regulatory challenge is therefore complex, immense. In 2008, when Health Canada initiated regulatory reform of natural health products, its public consultation received some of the largest public protests ever recorded. Petitioners argued, most notably, that regulatory efforts—indeed the very notion of regulation in this context—restricted and impinged upon individual freedom, signified government greed and corruption, and sought to unjustly restrict access to natural, safe, and effective products, perceived to be in stark contrast to harmful pharmaceuticals.^2^ It is facile to assume that regulatory bodies can effectively tackle the monsoon of online discourse promoting natural products and simultaneously generating fear, suspicion, and mistrust of science and health care systems. Regulatory bodies can and must, however, be bold, creative, industrious, and diligent to ensure regulatory mechanisms can effectively address inaccurate and harmful marketing. This may require critical reimagining of approaches and roles. Offering the veneer of regulatory oversight is not only wasteful but can further validate inaccurate and exploitative product claims among publics desiring accurate, evidence-based assessments from their governing leaders.

## Contributors

ARM wrote and edited the manuscript.

## Declaration of interests

The author has no conflicts of interest to declare.
